# Physical Analysis and Mathematical Modeling of the Hydrogen Storage Process in the MmNi_4.2_Mn_0.8_ Compound

**DOI:** 10.3390/ma17102237

**Published:** 2024-05-09

**Authors:** Sihem Belkhiria, Abdulrahman Alsawi, Chaker Briki, Saleh M. Altarifi, Mohamed Houcine Dhaou, Abdelmajid Jemni

**Affiliations:** 1 Laboratory of Thermal and Energy Systems Studies, University of Monastir, LR99ES31, Monastir 5019, Tunisia; 2 Department of Physics, College of Science, Qassim University, Buraidah 51452, Saudi Arabia

**Keywords:** hydrogen storage, storage (materials), isotherms, temperature, pressure

## Abstract

The results of an experimental and mathematical study into the MmNi_4.2_Mn_0.8_ compound’s hydrogen storage properties are presented in the present research. Plotting and discussion of the experimental isotherms (P-C-T) for different starting temperatures (288 K, 298 K, 308 K, and 318 K) were carried out first. Then, the enthalpy and entropy of formation (ΔH_0_, ΔS_0_) were deduced from the plot of van’t Hoff. Following that, the P-C-T were contrasted with a mathematical model developed via statistical physics modeling. The steric and energetic parameters, such as the number of the receiving sites (n_1_, n_2_), their densities (N_m1_, N_m2_), and the energy parameters (P_1_, P_2_) of the system, were calculated thanks to the excellent agreement between the numerical and experimental results. Therefore, plotting and discussing these parameters in relation to temperature preceded their application in determining the amount of hydrogen in each type of site per unit of metal ([H/M]_1_, [H/M]_2_) as well as for the entire system [H/M] versus temperature and pressure besides the absorption energies associated with each kind of site (ΔE^1^, ΔE^2^) and the thermodynamic functions (free energy, Gibbs energy, and entropy) that control the absorption reaction.

## 1. Introduction

The use of natural resources is essential to the advancement of both human society and the global economics. According to economists, it is imperative that energy consumption continues to rise for the purpose of advancing economic, cultural, and social development [1]. For example, to achieve a high level of development, the energy consumption per person must be approximately 33% higher than current global consumption [2]. However, the energy tactics used have a significant impact on inequalities in human development [3]. Currently, petroleum-based fuels such as coal, oil, and gas from natural sources provide approximately 95% of the energy required by the world. According to estimates, fossil fuel usage reached 3.3 terawatts (TW) in 2020, up 32% from the year 2000 [4].

Thus, even though fossil fuels still account for the majority of primary energy consumption today, countries all over the world have made the strategic decision to support the consumer revolution and the energy transformation in addition to developing a sustainable, solid, and environmentally friendly energy system [5,6,7,8].

Hydrogen is a promising alternative to existing power storage techniques that will be used in constructing our future energy system, including flywheels, power sources, air-compressed pumped water, capacitors that store energy, and others [9]. Thus, hydrogen is a feasible green energy vector due to its clean combustion characteristics, suitability for fuel cell vehicles, and potential energy, which is two-to-three times more performant than gasoline [10]. Nevertheless, before the hydrogen sector can see astronomical growth, a number of challenges must be overcome, just like with most renewable energy sources. These challenges usually relate to its expensive production, complicated storage, and large-scale use [11]. Therefore, storage is a crucial step toward hydrogen energy carriers [12]. Intrinsically, for commercial and practical uses, hydrogen storage systems must combine energy efficiency, security, and affordable storage costs [13]. One of the most promising storage technologies is the metal hydride form. Metal hydrides offer an appealing combination of high energy densities, safety, and numerous potential uses on both a small and large scale as well as the short and long term [14,15,16,17,18,19].

AB_5_ compounds stand out among the large family of hydrides as highly intriguing metal hydride candidates with very interesting hydrogen storing features since they have a significant storage capacity and safe operating conditions defined by moderate pressure and ambient temperature [20,21,22]. In reality, a hydrogen storage material needs to have a large hydrogen storage capacity, simple reversibility of the reactions leading to its formation and decomposition, and a low overall hydride cost (raw materials, manufacturing, and processing) for the application in question. It is also necessary to guarantee the long-term presence of natural materials (i.e., metallic resources) [23]. The hydride system (including its protection) should have the lowest possible cost per unit of hydrogen that can be stored reversibly. Other factors to consider include a storage vessel’s moderate cost, the cost of auxiliary equipment, the cost of fabrication and installation, and the cost of purchased energy that is not derived from waste or ambient air per storage cycle [24]. On top of that, AB_5_-based compounds continued to receive attention from researchers who are primarily interested in reaction kinetics, cycle stability, and material cost reduction [25,26,27,28,29,30,31,32,33,34,35,36,37].

For instance, the expensive lanthanum in LaNi_5_ can be substituted with less expensive rare earth elements like Ce [38,39] or a less expensive rare earth alloy made of Ce, Pr, Nd, and La known as mischmetal (Mm), which has been the subject of numerous investigations [40,41]. The compositional modifications have resulted in a 30% reduction in raw material prices when compared to the LaNi_5_ alloy [42]. Thus, the MmNi_5_ compound has demonstrated intriguing hydrogen storage characteristics, such as straightforward activation [42], elevated stability in the electrode potential [40], and a significant hydrogen storage capacity at room temperature, reaching 1.5% Wt [43].However, the MmNi_5_ compound has an equilibrium pressure of up to 30 bar [44]. This is a significantly higher value than, for instance, the equilibrium pressure of LaNi_5_, which is limited to 2 bar [45].Given that battery cells clearly operate at atmospheric pressures [46], this suggests a challenging engineering design for gas hydrogen storage, which was totally unworkable in MmNi_5_ to be utilized as the anode in a Ni-MH battery cell. In addition, despite the fact that MmNi_5_ is highly desirable for stationary applications, there are certain inherent limitations that make it impractical to use. One such limitation is the disproportion that occurs during subsequent hydrogen cycles, which is a serious problem since it reduces the hydrogen storage capacity during cycling [47].

Thus, in order to improve the hydrogen storage properties of the compound MmNi_5_, doping on the Mm and Ni sites is a promising strategy adopted by several researchers [48]. To accomplish this, a variety of metals have been employed, either separately or in combination like Al, Fe, Mn, etc. [45,49,50,51,52,53,54]. These studies showed that adding a particular dopant element allows for the improvement of the stability of hydrides, the prevention of decomposition, improving the properties of storing hydrogen, and the optimization of the thermodynamic characteristics of the MmNi_5_ hydride. For these reasons, we decided to consider a manganese-doped compound in the present investigation. Doping a compound like MmNi_5_ with additional manganese is a common strategy for hydrogen storage [55]. Compared to other doping elements, these compounds exhibit fast activation behavior, good corrosion resistance, moderate stability, a noticeable reaction enthalpy, and high catalytic activity [42,56,57,58]. Manganese hydrides are promising compounds that can store hydrogen reversibly in ambient conditions, according to even recent research [59]. In many energy storage applications such as transport and portable devices, its gravimetric and volumetric storage capacity allows it to compete with batteries while significantly reducing the supporting infrastructure’s complexity and cost [60]. The battery’s affordability, longevity, safety, dependability, sustainability, usability, and power or energy makes it a promising material for electric vehicle storage technologies [61]. Manganese doping is a commonly employed technique in metal hydrides to enhance their efficiency in various mobile and stationary applications by improving the ability of reversible storage of hydrogen [19]. As an example, the reduced rate of Mn, the simpler activation, and the decrease in the equilibrium pressure (for desorption, with 0.41 MPa to 0.26 MPa at 25 °C) with a marginally lower hysteresis (from 0.64 to 0.62 MPa) have all contributed to the Mn substitution’s increased appeal in hydrides composition [62,63,64].

Therefore, in light of the previous findings, the current study investigates the hydrogen absorption isotherms for the compound MmNi_4.2_Mn_0.8_ at different temperatures using both experimental and numerical methods. We decided to use a mathematical model grounded in statistical physics formalism [65]. This model has been widely used by researchers in the study of hydrogen storage processes [43,66,67,68,69,70,71,72,73]. The mathematical model expressions are susceptible to adjusting the experimental isotherms, making it possible to finely define the microscopic states of the absorption reaction. Thus, applying the statistical physics model to investigate the hydrogen absorption phenomenon is quite interesting. Firstly, as it contains in its expressions important physical parameters different from empirical models like Langmuir and Freundlich, whose parameters typically do not obtain a direct physical meaning that can be inferred from experimental data after a numerical simulation [66,67], the storage system can be investigated by determining the various physicochemical parameters related to the absorption phenomenon. Secondly, it provides a clear knowledge of the thermodynamic properties that control the evolution of the system during the absorption reaction, such as the reaction’s internal energy and entropy. The absorption isotherms were thus simulated using empirical models based on theoretical and mathematical expressions, formalized within the framework of the grand canonical ensemble of statistical physics. In a recent study [67], we investigated the hydrogen absorption characteristics of the compounds MmNi_4.6_Fe_0.4_ and MmNi_4.6_Al_0.4_ using this model. In this study, we experimentally and numerically investigated the effect of Mn doping on hydrogen storage compounds’ properties as a function of temperature and pressure. Thus, an intriguing hydrogen storage material, MmNi_4.2_Mn_0.8_, is reported in this study.

Such a model was used to calculate the energy coefficients (P_1_, P_2_), the interstitial site densities (N_m1_, N_m2_), and the number of atoms of hydrogen per site (n_1_, n_2_). Relevantly, the parameters acquired were then be applied to ascertain the thermodynamic functions that characterize the metal’s reactions to the absorption of hydrogen. Depending on the variations in pressure and temperature, the variation in hydrogen concentration per unit of metal (H/M), the internal energy (E_int_), the Gibbs free energy, and entropy (S) were be computed numerically and are discussed herein.

## 2. Equipment and Procedures

The Indian mixed metal Mm that was utilized has a typical composition of roughly 23% lanthanum, 18% neodymium, 43% cerium, 5% praseodymium, 8% iron, and 3% samarium. It was fused in an argon atmosphere with manganese and high-purity nickel (99.99%).

**Figure 1:**
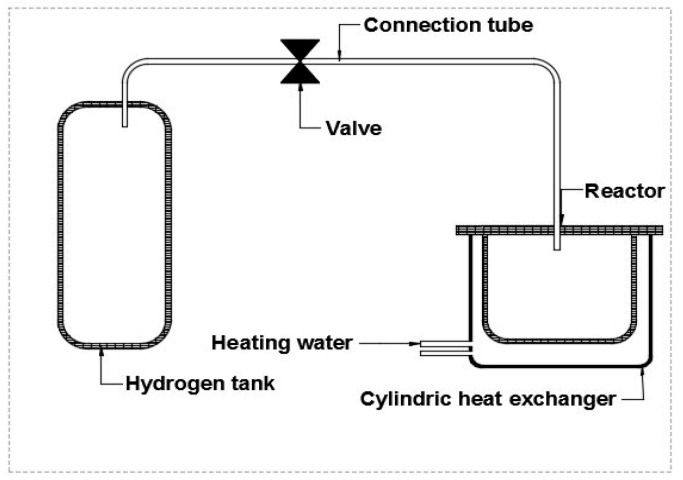
Experimental device.

The volumetric method was used to measure hydrogen absorption isotherms [68]. Its principle is as follows: A metal hydrogen reactor (MHR) with high pressure was filled with 20 g of the prepared material and connected to a hydrogen tank of buffer volume via Swagelok valves and aluminum connection tubes. During absorption, the hydrogen tank was charged to the desired pressure (P_0_) and placed in contact with the reactor. The hydrogen pressure decreased in the tank until it reached an equilibrium value (P_eq_). A pressure sensor was installed within the hydride bed, coupled with an Agilent acquisition card and a microcomputer, allowing the control of the temporal evolution of the pressure of the absorbed hydrogen. The temperature inside the hydride bed was regulated by cooled water from a thermostatic bath through an external cylindrical heat exchanger (Figure 1).

## 3. Experimental Isotherms

**Figure 2:**
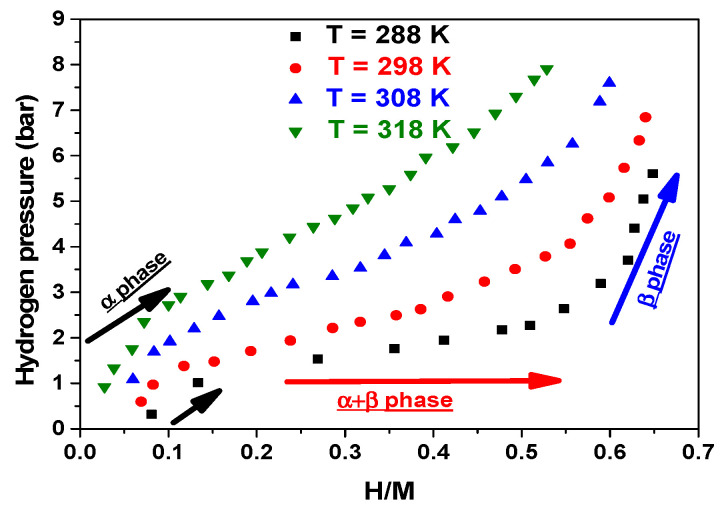
Absorption isotherms in the MmNi_4.2_Mn_0.8_ compound at different temperatures.

Typically, P-C-T isotherms, which show the pressure–concentration–temperature records, are monitored to characterize a metal hydride system. Figure 2 presents the experimental isotherms of the absorption of hydrogen by MmNi_4.2_Mn_0.8_ versus different temperatures.

The hydrogen capacity, i.e., concentration, is commonly expressed in terms of atoms of hydrogen per metal species, i.e., H/M. It is most effective to use the its highest capacity [H/M]_max_ to characterize the metal hydride. The following could serve as a summary of the key findings pertaining to the acquired outcomes. It is seen from Figure 2 that at a given temperature, an isotherm is composed of three phases:

* α phase: distinguished by a sharp rise in pressure against a slight change in H/M;
* α + β phase: marked by a remarkable increase in H/M and a slight shift in pressure (equilibrium plateau);
* β phase: characterized by a stabilization of H/M against a rise in pressure.

Actually, atoms of hydrogen in the α phase of the crystal lattice are in solid solution at low-hydrogen compositions. The system’s hydrogen content affects the equilibrium pressure of this phase. As the temperature rises, the α-phase saturation rate increases. Then, at higher hydrogen rates, a α-phase structural transition gives rise to the β phase (rutile structure), a hydride with a specific composition. Constant pressure causes this transformation to occur. Thus, the presence of the phases α and β together occurs on an equilibrium plateau up to a rate that is not too far from the β phase’s saturation. The majority of the hydrogen is absorbed in the plateau region, with a slight change in pressure. This plate’s pressure rises as its temperature does. Beyond this, saturating the β phase requires a significant increase in pressure. Thus, the amount of hydrogen absorbed above the plateau pressure is extremely small.

Therefore, a hydride storage material’s equilibrium dissociation pressure (P_eq_) is one of its most crucial characteristics. According to Figure 2, if the temperature changes between 288 K and 318 K, the equilibrium plateau of the compound MmNi_4.2_Mn_0.8_ is situated roughly between 2 bar and 5 bar. We must consider that, for instance, the equilibrium pressure of the compound MmNi_4.2_Mn_0.8_ at 218 K (5 bar) is essentially half of that of the compound La_0.91_Ce_0.9_Ni_5_ (8 bars) at the same experimental conditions and practically of the same order of magnitude as that of LaNi_5_ (5 bar) [43]. Furthermore, the Mn-doped compound’s equilibrium pressure is nearly half that of the MmNi_5_ compound [69]. Likewise, the Mn-doped compound is at roughly the same equilibrium plateau level as the compound MmNi_4.6_Al_0.4_ and shows an equilibrium plateau drop that is roughly five times smaller than that of the Fe-doped compound [67]. In view of this, MmNi_4.2_Mn_0.8_ is thought to have a lower and more promising equilibrium pressure. As a result, the compound presents the simple reversibility, cyclability, and stability of the hydride under cycling at room temperature.

Without a doubt, there is still less than one hydrogen atom for every metal unit. In comparison, at a given temperature, the Fe-doped compound has almost twice the H/M of the Mn-doped compound. For example, at 288 K, the MmNi_4.2_Mn_0.8_ compound shows an [H/M] max of 0.6, while the compound MmNi_4.6_Fe_0.4_ exhibits an [H/M] _max_ of 1.2 [67].

## 4. The Plot of Van’t Hoff

**Figure 3:**
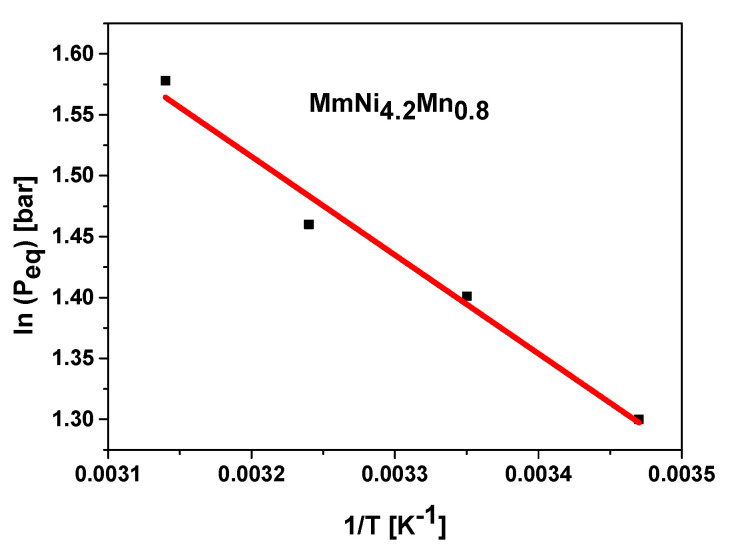
The plot of van’t Hoff.

Plotting the equilibrium pressure (P_eq_) versus the reciprocal of the absolute temperature (1/T) is referred to as the van’t Hoff plot. The standard enthalpy (ΔH_0_) and entropy (ΔS_0_) of the reaction can be determined using the slope and the intercept of the plot, as shown in the van’t Hoff equation [70]:

(1) $$Ln\left(\frac{P_{0}}{P_{eq}}\right)=\frac{\Delta H_{0}}{RT}-\frac{\Delta S_{0}}{R}$$

where T is the absolute temperature, R is the constant of ideal gas, and P_0_ is the standard pressure, which is usually taken to be 1 atm. Figure 3 presents the van’t Hoff plot corresponding to MmNi_4.2_Mn_0.8_ at the investigated temperature.

The absorption reaction’s enthalpy and entropy are then inferred from the plot. Table 1 provides their values.

The quantity of heat that is let out during the absorption of hydrogen and that must be supplied again during desorption is determined by the formation enthalpy. This value is in good agreement with that of type-AB_5_ alloys [71]. The fact that ΔH is negative indicates that the reaction of absorption is exothermic, as the hydride bed system releases heat during this process. Also, ΔS decreases as a result of the change from a more disordered gaseous state (gaseous hydrogen) to a more ordered solid state (hydride), as indicated by the negative value of entropy.

## 5. Modelling

### 5.1. Introduction of the Model

The double-energy mono-layer model with two types of sites is a mathematical model developed by statistical physics. Thus, it is the most stable among the statistical physics-based numerical models since it most closely matches the experimental absorption data, as indicated by Bouaziz N. et al. [72]. Therefore, this model was used in the present study. When using a model, a number of considerations and assumptions must be made [73]. Firstly, hydrogen must be considered an ideal gas (at low pressure). Moreover, we must only consider the two most important degrees of freedom for atoms: translation with a typical translational temperature of φ_tr_ = 10^−15^ K and rotation with a typical rotational temperature of φ_rot_ = 85.3 K. Furthermore, remember that a varying amount of n_a_ atoms of hydrogen is stored in N_m_ interstitial sites spaced across the unit mass of the absorbent material. Thereby, the equation describing the reversible hydrogen storage reaction in the metal is as follows (Equation (2)):

(2) $$M+\frac{n}{2}H_{2}⇌MH_{n}$$

In the following reaction, M presents the hydrogen storage material, n presents the stoichiometric coefficient, and MH_n_ presents the formed hydride. Moreover, the hydrogen atoms in metal alloys can be viewed as aggregated in (H_n_).

Since the sorption process involves a transfer of particles from the state of freedom to the absorption state, the grand canonical ensemble must be utilized to study it (Equation (3)):

(3) $$z_{gc}=\sum_{N_{j}}e^{-\beta (-\varepsilon_{j}-\mu )N_{j}}$$

where N_j_, the occupation state of the receptor sites, is either equal to 0 or 1; −(−εj) denotes the absorption energy at the receptor sites; µ, the absorbed site’s chemical potential, is equivalent to 1/(K_B_T), where k_B_ denotes the Boltzmann constant. The system’s microscopic states are defined by the grand canonical partition function (Z_gc_) in relation to the physical circumstances in which it is situated. Given that, the N_m_ receptor sites per mass unit are linked to the total grand canonical partition function. In the event that these sites are considered to be independent and identical, Z_gc_ can be represented as a simple product (Equation (4)):

(4) $$Z_{gc}=\prod_{j}{\left(Z_{gc}\right)}^{N_{jm}}$$

As a result, two atoms of hydrogen, N_1_ and N_2_, are stored in two distinct types of interstitial sites (n_1_ and n_2_) in the monolayer model with two energies. The density in the first category of sites is N_m1_, while the density in the second category is N_m2_. The energies ε_1_ and ε_2_, respectively, define them. Under these circumstances, Z_gc_ can be written as (Equation (5)):

(5) $$Z_{gc}={\left(z_{1gc}\right)}^{N_{1m}}\times {\left(z_{2gc}\right)}^{N_{2m}}$$

Z_gc_ of the first and second site types are denoted by Z_1gc_ and Z_2gc_, respectively:

(6) $$z_{1gc}=\sum_{Nj=0,1}e^{\frac{(-\varepsilon_{1}-\mu )N_{j}}{k_{B}T}}=1+e^{\beta (\varepsilon_{1}+\mu )}$$

(7) $$z_{2gc}=\sum_{Nj=0,1}e^{\frac{(-\varepsilon_{2}-\mu )N_{j}}{k_{B}T}}=1+e^{\beta (\varepsilon_{2}+\mu )}$$

The chemical potential of an individual free dihydrogen molecule is expressed in the gaseous form as (Equation (8)):

(8) $$\mu_{m}=\mu /n=k_{B}T\ln\left(\frac{N}{z_{gc}}\right)$$

where µ represents the chemical potential of the interstitial site, and n denotes the number of atoms per site. Z_gc_ can be expressed as follows (Equation (9)):

(9) $$z_{gc}=z_{gtr}\times z_{grot}=V{\left(\frac{2\pi mk_{B}T}{h^{2}}\right)}^{3/2}*\frac{T}{2\theta_{rot}}$$

where Z_grot_ and Z_gtr_ stand for the translation and rotation partition functions, respectively. The parameters of the system are as follows: m is the mass of a hydrogen atom, V is its volume, h is Planck’s constant (h = 6.6260 × 10^−34^ m^2^ kg/s), and θ_rot_ is the dihydrogen molecule’s characteristic rotational temperature. For an ideal hydrogen gas, z_gtr_ can be expressed using the expression in relation to the saturated vapor pressure P_VS_ and the vaporization energy (ΔE_v_) as follows (Equation (10)):

(10) $$Z_{gtr}=\beta P_{vs}e^{\Delta E_{V}}z_{gtr}=\beta P_{vs}e\frac{\Delta E_{v}}{RT}$$

where

(11) $$P_{VS}={\left(\frac{2\Pi K_{B}T}{h^{2}}\right)}^{\frac{3}{2}}K_{B}Te\frac{-\Delta E_{v}}{RT}$$

Therefore, using Equation (2), the following formulae yield the average numbers of occupied sites (Equation(12)):

(12) $$N_{O}=n_{1}N_{01}+n_{2}N_{02}=n_{1}\frac{N_{1m}}{1+{\left(\frac{P_{1}}{P}\right)}^{n_{1}}}+n_{2}\frac{N_{2m}}{1+{\left(\frac{P_{2}}{P}\right)}^{n_{2}}}$$

where P_1_ = $K_{B}$T$Z_{g}e^{-{\;\;}_{1m}}$ and P_2_ = $K_{B}$T$Z_{g}e^{-{\;\;}_{2m}}$ are the saturation pressures in the middle, which identify the first and second site types, respectively.

We must consider that the amounts that are experimentally stored are expressed in terms of H/M or the number of H atoms per unit formula. Thereby, for every type of site, the absorbed amount is equal to (Equation (13)):

(13) $${\left[\frac{H}{M}\right]}_{1}=\frac{n_{1}N_{1m}}{\left(N_{1m}+N_{2m}\right)\left(1+{\left(\frac{P_{1}}{P}\right)}^{\left(\frac{n_{1}}{2}\right)}\right)}=\frac{{\left[\frac{H}{M}\right]}_{1sat}}{1+{\left(\frac{P_{1}}{P}\right)}^{\left(\frac{n_{1}}{2}\right)}}$$

(14) $${\left[\frac{H}{M}\right]}_{2}=\frac{n_{2}N_{2m}}{\left(N_{1m}+N_{2m}\right)\left(1+{\left(\frac{P_{2}}{P}\right)}^{\left(\frac{n_{2}}{2}\right)}\right)}=\frac{{\left[\frac{H}{M}\right]}_{2sat}}{1+{\left(\frac{P_{2}}{P}\right)}^{\left(\frac{n_{2}}{2}\right)}}$$

with

$$\left[\frac{H}{M}\right]={\left[\frac{H}{M}\right]}_{1}+{\left[\frac{H}{M}\right]}_{2}\;\mathrm{and}\;{\left[\frac{H}{M}\right]}_{sat}={\left[\frac{H}{M}\right]}_{1sat}+{\left[\frac{H}{M}\right]}_{2sat}$$

Subsequently, the following defines the equation for the total quantity absorbed or desorbed per unit formula:

(15) $$\left[\frac{H}{M}\right]=\frac{{\left[\frac{H}{M}\right]}_{1sat}}{1+{\left(\frac{P_{1}}{P}\right)}^{\left(\frac{n_{1}}{2}\right)}}+\frac{{\left[\frac{H}{M}\right]}_{2sat}}{1+{\left(\frac{P_{2}}{P}\right)}^{\left(\frac{n_{2}}{2}\right)}}$$

In order to interpret the absorption process, six fitted parameters were determined for this model: the atoms numbers per site for each type (n_1_, n_2_), their densities of (N_m1_, N_m2_), and the pressures at middle saturation (P_1_ and P_2_).

Note: Absorption involves the penetration of hydrogen atoms into the volume of the storage material, while adsorption is the state that occurs on the surface of the substrate. Several studies have considered that absorption and adsorption are energetically similar since both mechanisms belong to the same category of diffusional equilibrium phenomena. Thus, several researchers have shown that the double-energy mono-layer model is suitable with excellence for studying the phenomenon of hydrogen absorption by metal hydrides [71,72,73].

### 5.2. Adjustment of the Experimental P-C-T

**Figure 4:**
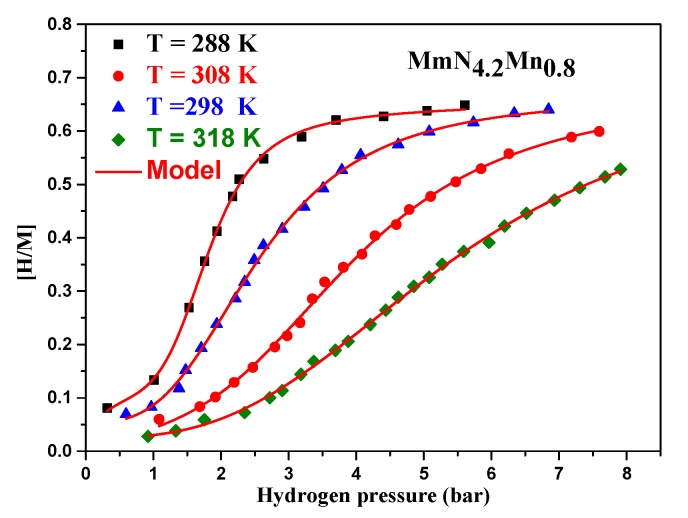
Fitting of the absorption isotherms using the double-energy mono-layer model.

Using the model previously described, the hydrogen absorption isotherms were adjusted. The adjustment demonstrates that the suggested model and the experimental data agree perfectly (Figure 4).

Depending on the model selected, we can explain the development of the two distinct phases: a first hydride solid solution, which is thought to be phase α, and a second hydride phase, which is thought to be phase β. As a result, the two-energy mono-layer model that was employed to fit the experimental data makes the reaction easier to study and accurately depicts how hydrogen is reversibly stored by the compound. Table 2 displays the values of the fitting parameters versus temperature during the absorption and desorption processes.

Prior to being utilized to determine the thermodynamic functions characterizing the hydrogen storing reaction, such as Gibbs free energy, the internal energy, and entropy, these parameters will be examined as a function of temperature.

### 5.3. Numerical Findings

#### 5.3.1. Temperature’s Effect on n_1_, n_2_, and n

The number of sites available for hydrogen storage in hydride form and temperature have a complex relationship that depends on the particulars of the hydride material. The statistical physics model proposed for modeling the hydrogen storage in MmNi_4.2_Mn_0.8_ requires the existence of two types of interstitial sites to receive or release hydrogen atoms during absorption or desorption. In these circumstances, Equation (1) can be viewed as the sum of the following two equations that show the contributions of n_1_ and n_2_, which represent the number of hydrogen atoms in the first and second types of sites, respectively:

(16) $$M+\frac{n_{1}}{2}↔M_{1}H_{n_{1}}$$

(17) $$M+\frac{n_{2}}{2}↔M_{2}H_{n_{2}}$$

(18) $$M+\frac{n_{1}+n_{2}}{4}H_{2}↔MH_{\frac{n_{1}+n_{2}}{2}}$$

From Equations (1) and (12), it can be deduced that n can be expressed as follows:

(19) $$n=\frac{n_{1}+n_{2}}{2}$$

**Figure 5:**
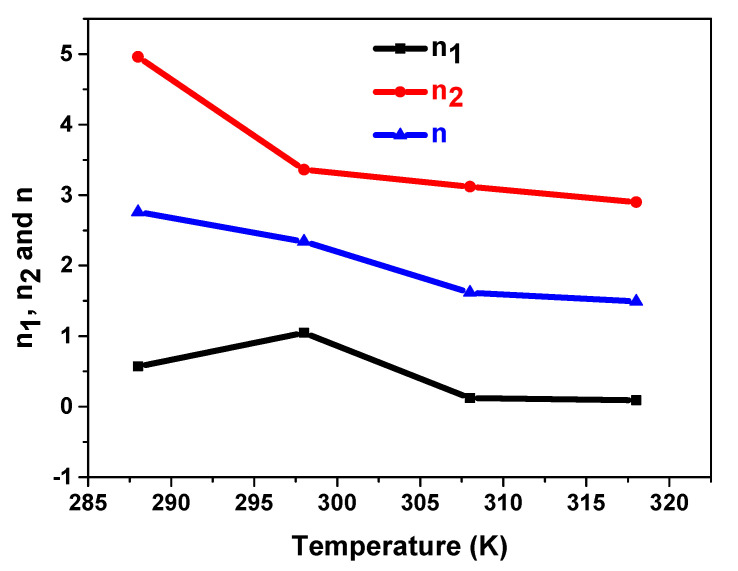
Dependence of n_1_, n_2_, and n on temperature during hydrogen absorption in the compound MmNi_4.2_Mn_0.8_.

The variables n_1_, n_2_, and n are given in Figure 5.

As seen, the two kinds of sites behave differently depending on temperature and do not have the same order of magnitude. Firstly, the result is n_1_ < n_2_. The distinction in size between the first-phase and second-phase sites could provide justification for this aspect.

Then, it is shown that, as the temperature rose from 288 K to 298 K, there were more hydrogen atoms in the first type of site n_1_. A decrease in the quantity of hydrogen atoms in the second class of sites (n_2_) offsets this increase. Afterward, the quantity of hydrogen atoms in the two types of sites decreases as the temperature rises. As an outcome, n decreases linearly as a result of n_1_ and n_2_’s reactions to the temperature increase. The exothermic character of the reaction of hydrogen absorption explains the decrease in n with temperature. As a result, as demonstrated, the rise in temperature blocks the interstitial receptor sites for hydrogen atoms, which typically results in a hindrance to the absorption reaction. Another point that needs to be noted is that since n is still greater than 1, it is evident that there are multiple hydrogen atoms at each site. This suggests that the interstitial sites of the metal matrix experience a phenomenon of hydrogen atom agglomeration.

#### 5.3.2. Temperature’s Influence on Interstitial Site Densities N_m1_ and N_m2_

**Figure 6:**
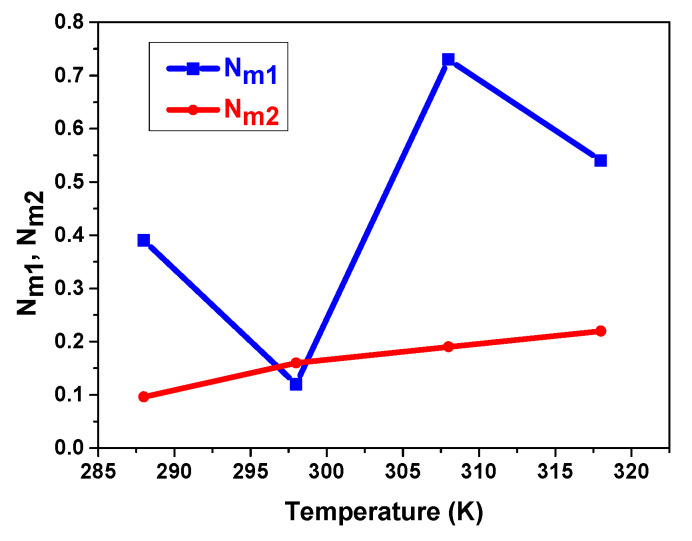
Dependence of N_m1_ and N_m2_ on temperature during hydrogen absorption in the compound MmNi_4.2_Mn_0.8_.

For sites type 1 and type 2, N_m1_ and N_m2_ reveal the site number actually needed to absorb the number of atoms at saturation. Figure 6 depicts the temperature dependence of N_m1_ and N_m2_.

Firstly, it is shown that during absorption, N_m1_ > N_m2_. This indicates that there are more sites in the first phase than in the second phase of the crystalline lattice of the MmNi_4.2_Mn_0.8_ hydride. Secondly, it is demonstrated that N_m1_ and N_m2_ interact differently depending on the temperature. Therefore, a notable fluctuation of N_m1_ is observed despite the fact that the variation of N_m2_ with temperature is limited. This may lead to pulverization and degradation, potentially destroying the hydride’s metal matrix. In fact, the simple destructibility of the substrate’s surface brought on by volume expansion and the fragility of metal alloys are the main causes of their degradation. As a result, considerable fracturing, an intense gradient generating defects from the sample’s surface towards the mass, and some cracks on the alloy’s surface are seen during hydrogen absorption [73].

#### 5.3.3. Temperature’s Influence on [H/M]_1sat_ and [H/M]_2sat_

As previously demonstrated (Equation (12)) using the proposed model, the total absorption can be analyzed by two distinct contributions. Therefore, for the first and second types of sites, the absorbed amounts per unit formula at saturation are determined by (n_1_, N_m2_) and (n_2_, N_m2_), respectively, as follows:

(20) $${\left[\frac{H}{M}\right]}_{1sat}=\frac{n_{1}N_{m1}}{N_{m1}+N_{m2}}$$

(21) $${\left[\frac{H}{M}\right]}_{2sat}=\frac{n_{2}N_{m2}}{N_{m1}+N_{m2}}$$

**Figure 7:**
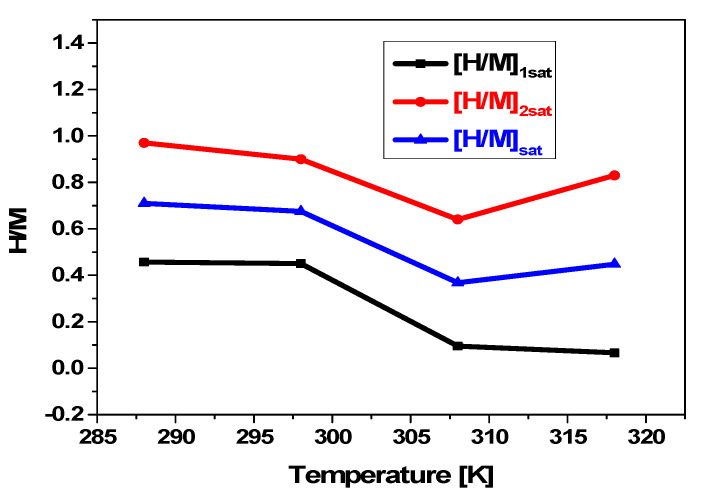
Dependence of [H/M]_1sat_, [H/M]_2sat_, and [H/M] _sat_ on temperature during hydrogen absorption in the compound MmNi_4.2_Mn_0.8._

The evolution of [H/M]_1sat_, [H/M]_2sat_, and [H/M] _sat_ of the absorption reaction depending on temperature is depicted in Figure 7.

First, [H/M]_1sat_ < [H/M]_2sat_ is demonstrated. The fact that n_2_ > n_1_ explains this.

Then, for both kinds of sites, it is obvious that as temperature rises, the maximum amount of hydrogen absorbed falls. Consequently, the absorption process is exothermic for both kinds of sites as well as for the global system marked by [H/M]. Thus, thermal agitation is the cause of these variations. The experimental values (Figure 1) and the numerical values (Figure 7) of [H/M] as a function of temperature agree exactly.

#### 5.3.4. Temperature’s Influence on the ΔE^1^ and ΔE^2^

The heat of absorption can be used to describe hydrogen absorption energies. These parameters are critical in determining the nature of the interaction between hydrogen and the MmNi_4.2_Mn_0.8_ compound. The energies of hydrogen absorption (ΔE^1^ and ΔE^2^) for each type of site were calculated using the following formula:

(22) $$\Delta E^{1}=RT\ln\left(\frac{P}{P_{1}}\right)$$

(23) $$\Delta E^{2}=RT\ln\left(\frac{P}{P_{2}}\right)$$

where P is expressed in the following format:

(24) $$P=\exp\left[12.69-\frac{94.896}{T}+\left(1.1125\right)\ln\left(T\right)+\left(3.2915\times 10^{-4}\right)T^{2}\right]$$

**Figure 8:**
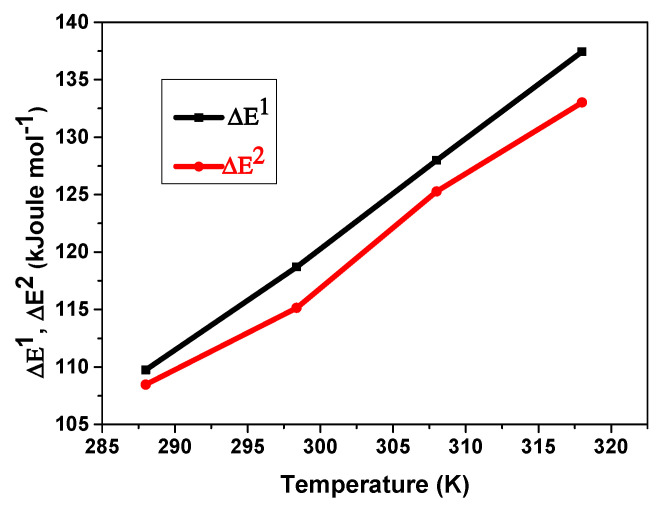
Dependence of ΔE^1^ and ΔE^2^ on temperature during hydrogen absorption in the compound MmNi_4.2_Mn_0.8._

According to Figure 8, which depicts the relationship between the temperature and the energies of absorption, both ΔE^1^ and ΔE^2^ rise nearly linearly with rising temperatures with nearly equal values. This implies that at higher temperatures, greater pressure is needed for hydrogen to be transferred from the gaseous form to the absorbed phase. This is consistent with the hydrogen absorption reaction’s exothermic nature. This is explained by the absorption reaction’s exothermic character. Moreover, the calculated energies range between 107 and 137 Kj/mol. This demonstrates that hydrogen atoms are chemically bound to the two types of receptor sites. The difference in ΔE^1^ and ΔE^2^ is the root cause of the difference in the first and second type of site sizes.

#### 5.3.5. Variation in Hydrogen Concentration [H/M] with Pressure at Different Temperatures

**Figure 9:**
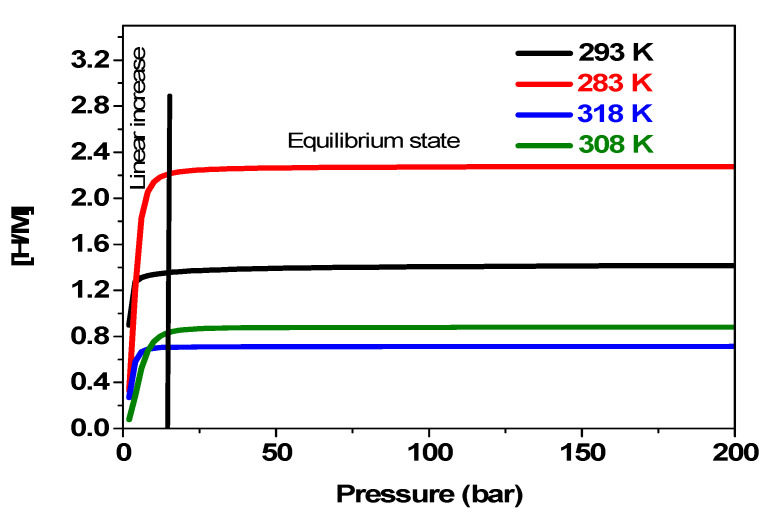
Variation of [H/M] depending on the applied pressure at 283 K, 293 K, 308 K, and 318 K.

Pressure affects the process of hydrogen absorption significantly, just like temperature does. It is one of the most important factors to take into account when developing and perfecting hydrogen storage systems. The [H/M] variation as a function of applied pressure versus temperature is shown in Figure 9.

As a starting point, we find from Figure 9 that raising the initial temperature decreases both the kinetics of the reaction and the amount of hydrogen absorbed, resulting in a less efficient reaction since the absorption reaction is exothermic. For instance, the maximum concentration of hydrogen per unit of metal decreases by about three times when the temperature rises from 288 k to 318 k. Additionally, it is demonstrated that at a given temperature, until the stabilization that occurs in the equilibrium state, the absorbed amount increases gradually depending on pressure. Therefore, hydrogen molecules occupy an increasing number of sites on the material as the pressure rises. The maximum amount of hydrogen that can be adsorbed, denoted by [H/M] max, is typically the upper limit. The absorption process may get close to saturation, and the amount of hydrogen adsorbed may stabilize or plateau at higher pressures. When the pressure no longer significantly increases the amount of hydrogen adsorbed, the equilibrium state is reached.

#### 5.3.6. Effects of Pressure on the System’s Internal Energy Variation U_int_ versus Temperatures

The total energy present in a system is represented by internal energy, i.e., U_int_, a fundamental and comprehensive function. It consists of potential energy, which is stored energy dependent on position or state, as well as kinetic energy, which is the energy of motion. The internal energy of a system is greatly influenced by its temperature and pressure. The following is the expression for the internal energy of hydrogen absorption [68]:

(25) $$U_{\mathrm{int}}=-\frac{\partial \ln(z_{gc})}{\partial \beta }+\frac{\mu }{\beta }(\frac{\partial \ln(z_{gc})}{\partial \mu })$$

with

(26) $$\mu =K_{B}T\ln\left(\frac{\beta P}{z_{g}}\right)$$

Consequently, U_int_ can be expressed in the following manner:

(27) $$\begin{array}{l} U_{\mathrm{int}}=k_{B}\ln(\frac{\beta P}{z_{g}})\left(\frac{N_{1m}{\left(\frac{P}{P_{1}}\right)}^{n_{1}}}{1+{\left(\frac{P}{P_{1}}\right)}^{n_{1}}}+\frac{N_{2m}{\left(\frac{P}{P_{2}}\right)}^{n_{2}}}{1+{\left(\frac{P}{P_{2}}\right)}^{n_{2}}}\right)\\ -k_{B}T\left(\frac{N_{1m}{\left(\frac{P}{P_{1}}\right)}^{n_{1}}}{1+{\left(\frac{P}{P_{1}}\right)}^{n_{1}}}+\frac{N_{2m}{\left(\frac{P}{P_{2}}\right)}^{n_{2}}}{1+{\left(\frac{P}{P_{2}}\right)}^{n_{2}}}\right) \end{array}$$

**Figure 10:**
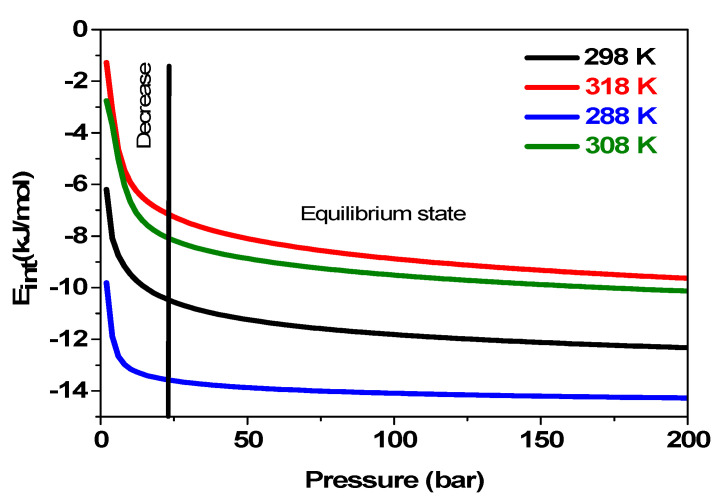
Variation of the U_int_ depending on the applied pressure at 283 K, 293 K, 308 K, and 318 K.

The variation of U_int_ depending on pressure versus various temperatures during the reaction of hydrogen absorption by the MmNi_4.2_Mn_0.8_ compound is shown in Figure 10.

Firstly, it is shown from Figure 10 that the system must supply energy since the internal energy values are negative, which prevents hydrogen atoms from being absorbed. This indicates that the process of absorption is exothermic. This clarifies why the system’s internal energy increases as a result of the initial temperature rising. For instance, the energy dissipated at 288 K is seven times less than that required to be supplied at 318 K. Moreover, it is noted that as pressure rises, the values of the internal absorption energies decrease. This suggests that at high pressure, the system can absorb hydrogen with less energy.

#### 5.3.7. Effects of Pressure on the System’s Entropy Variation versus Temperatures

Entropy is a thermodynamic function that quantifies the level of disorder or randomness in a system both prior to and following a chemical reaction. It shows how the system’s energy and matter distribution have changed. The grand potential and the absorption entropy are related by the following relationships:

(28) $$J=-K_{B}T\ln\left(z_{gc}\right)$$

(29) $$J=-\frac{\partial \ln\left(z_{gc}\right)}{\partial \beta }-TS_{a}$$

(30) $$TS_{a}=-\frac{\partial \ln\left(z_{gc}\right)}{\partial \beta }+K_{B}T\ln\left(z_{gc}\right)$$

Consequently, the entropy of the absorption Sa can be defined using the fitted parameters in this way:

(31) $$\begin{array}{l} S_{a}=-k_{B}\left(N_{1m}\ln\left(1+{\left(\frac{P}{P_{1}}\right)}^{n_{1}}\right)+N_{2m}\ln\left(1+{\left(\frac{P}{P_{2}}\right)}^{n_{2}}\right)\right)\\ -\left(\frac{N_{1m}{\left(\frac{P}{P_{1}}\right)}^{n_{1}}\ln{\left(\frac{P}{P_{1}}\right)}^{n_{1}}}{1+{\left(\frac{P}{P_{1}}\right)}^{n_{1}}}+\frac{N_{2m}{\left(\frac{P}{P_{2}}\right)}^{n_{2}}\ln{\left(\frac{P}{P_{2}}\right)}^{n_{2}}}{1+{\left(\frac{P}{P_{2}}\right)}^{n_{2}}}\right) \end{array}$$

**Figure 11:**
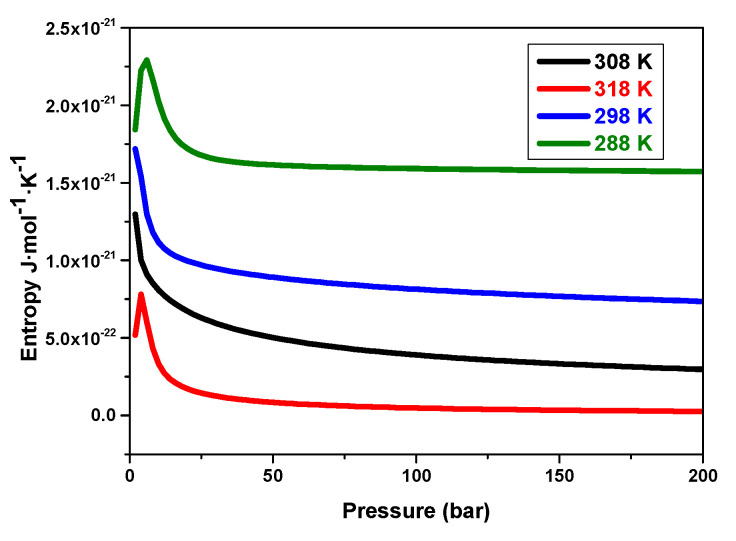
Variation of the entropy depending on the applied pressure for different temperatures.

Figure 11 presents the entropy variation of the reaction of absorption by the MmNi_4.2_Mn_0.8_ compound depending on pressure versus different temperatures.

Thefindings demonstrated that, at a specific temperature, the absorption reaction’s entropy crosses a maximum (a pic). Throughout the absorption reaction, the disorder progressively rises, reaches its highest point near the equilibrium state, and then eventually drops and maintains stability, indicating that the reaction has attained its state of equilibrium. The entropy’s maximum is found to decrease with rising temperatures. This can be attributed to the endothermic character of the reaction.

#### 5.3.8. Effects of Pressure on the Gibbs Variation versus Temperatures

The Gibbs free energy is expressed as follows:

(32) $$G_{a}=\mu \times n\times N_{0}=\mu {\left[\frac{H}{M}\right]}_{0}$$

As a result, Ga is written depending on the fitted parameters as given below:

(33) $$G_{a}=K_{B}\ln\left[\frac{\beta P}{z_{g}}\left(\frac{n_{1}N_{1m}}{1+{\left(\frac{P_{1}}{P}\right)}^{\left(\frac{n_{1}}{2}\right)}}+\frac{n_{2}N_{2m}}{1+{\left(\frac{P_{2}}{P}\right)}^{\left(\frac{n_{2}}{2}\right)}}\right)\right]$$

**Figure 12:**
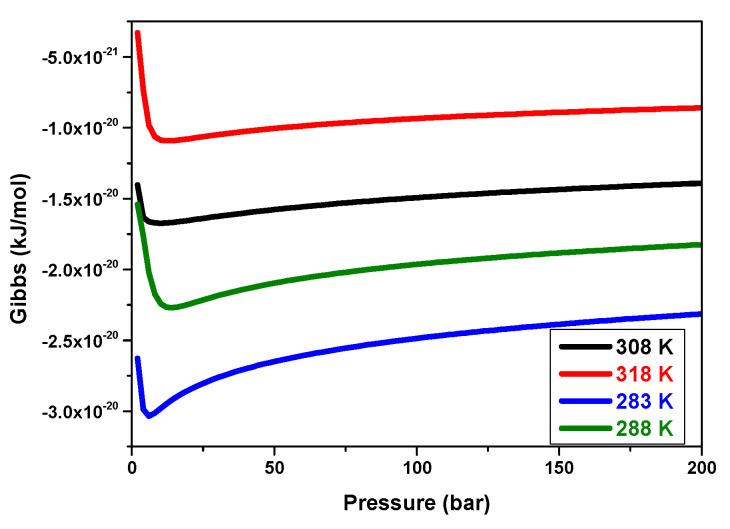
Gibbs free energy evolution for MmNi_4.2_Mn_0.8_ at various temperatures as a function of pressure.

The Gibbs variation versus pressure at various temperatures during hydrogen absorption by the MmNi_4.2_Mn_0.8_ compound is shown in Figure 12.

Firstly, the absorption phenomenon appears to be thermodynamically spontaneous in nature, as indicated by the negative values of the Gibbs energy. Then, as the temperature rises, the free enthalpy values rise as well, indicating that absorption is less likely to occur at high temperatures. The temperature makes it impossible for the absorption mechanism to be accomplished.

Note: Entropy and Gibbs free energy infer directly about the variation of the enthalpy H since the enthalpy change is the sum of the Gibbs free energy change and the product of the absolute temperature times the entropy change.

## 6. Conclusions

This article examined the properties of hydrogen absorption by the compound MmNi_4.2_Mn_0.8_ through both experimental and numerical modeling. Firstly, it was demonstrated by experimental hydrogen absorption isotherms that a lower and more promising equilibrium pressure results from doping Ni with Mn. This thus demonstrates the fundamental properties of cyclability, hydride stability, and simple reversibility under cycling at room temperature. Then, the experimental results were compared to a numerical model based on the formalism of statistical physics. Therefore, the characteristics of steric and energetic factors implicated in the hydrogen absorption reaction, including (n_1_, n_2_), (N_m1_, N_m2_), and (P_1_, P_2_), were determined and discussed depending on the temperature thanks to the perfect agreement between the experimental and numerical data. Ultimately, these parameters were utilized to translate a microscopic description of the absorption process into macroscopic characteristics. Consequently, the heat of the absorption reaction and the concentration of hydrogen atoms per unit of hydride were calculated. In addition, the three thermodynamic entropy, internal energy, and Gibbs free energy functions that control the hydrogenation process were measured and examined according to pressure variation at various operating temperatures. The results showed that increasing the temperature blocks the interstitial receptor sites n for hydrogen atoms, which generally results in an obstacle to the absorption reaction. Although the literature already contains the experimental outcomes, the theoretical instrument displays its resilience in characterizing the compound’s process of absorbing hydrogen.

## Figures and Tables

**Table 1:** The absorption reaction’s enthalpy and entropy values.

ΔH_0_ (kJ mol^−1^)	ΔS_0_ (J mol^−1^ K^−1^)
−12.4	−81.18

**Table 2:** Fitting parameters for the MmNi_4.2_Mn_0.8_ compound for the hydrogen absorption versus temperature.

Absorption Process							
Temperature (K)	n_1_	n_2_	n	Nm_1_	Nm_2_	P_1_	P_2_
**288**	0.57	4.96	2.76	0.39	0.096	1.05	1.8
**298**	1.05	3.63	2.34	0.12	0.16	2.47	3.99
**308**	0.12	3.12	1.62	0.73	0.19	0.04	2.55
**318**	0.093	2.9	1.49	0.54	0.22	1.01	5.43

## Data Availability

Data are contained within the article.
